# Developmentally programmed obesity: Is there a role for anti‐inflammatory nutritional strategies?

**DOI:** 10.1113/EP091209

**Published:** 2023-11-30

**Authors:** Michelle L. Kearns, Clare M. Reynolds

**Affiliations:** ^1^ Conway Institute/School of Public Health Physiotherapy and Sports Science/Institute of Food and Health/Diabetes Complications Research Centre University College Dublin Dublin 4 Ireland

**Keywords:** adipose tissue, anti‐inflammatory, early life, maternal diet, obesity

## Abstract

Pregnancy represents a period of immense maternal physiological adaptation, with progressive increases in lipid storage potential and insulin resistance to support fetal/placental growth. This requires significant change in the adipose tissue. Women living with obesity/overweight are more susceptible to these changes causing complications such as gestational diabetes. This is particularly worrying as up to 60% of European women are living with overweight/obesity at the onset of pregnancy. Furthermore, less than 1% meet all nutrition guidelines. There is now evidence that these deep metabolic changes can result in a predisposition to metabolic disease in both the mother and child in later life. Health and nutrition status during this period therefore represents a window to future health. This period offers a valuable opportunity for intervention to prevent the negative consequences of poor in utero environments and increases the long‐term quality of life for mother and offspring. This review will examine a range of in utero factors which determine adipose tissue development, the impact of these factors on later‐life obesity and metabolic health and the therapeutic value of dietary anti‐inflammatory nutritional interventions during pregnancy and early life. When it comes to early life nutrition, a ‘one size fits all’ approach is not always appropriate. Understanding the mechanisms of adipose tissue development in response to differing nutritional strategies may be important in the context of complicated or adverse in utero environments and represents a substantial step towards a more personalised nutritional approach for the prevention of obesity, metabolic syndrome and related non‐communicable diseases in future generations.

## Abstract (highlights)

- **What is the topic of this review?**
  The influence of adversein utero environments (obesogenic, undernutrition, gestational diabetes) onadipocyte capacity and the associated risk of obesity and metabolicdysregulation in later life.
- **What advances does it highlight?**
  Novel anti‐inflammatory dietary intervention strategies duringpregnancy that aim prevent obesity and metabolic dysfunction in the offspring. Despiteoverwhelming evidence of inflammatory involvement in obesity and associatedcomorbidities, the involvement of metabolic inflammation on pregnancy andearly‐life programming of metabolic disease has not been investigatedcomprehensively. Studies identifying novel intervention strategies to combat apoor start to life are pivotal in the developmental programming field given thedual approach of both identifying valid nutritional interventions while at thesame time aiding in the identification of molecular targets that influenceadipose tissue function early in the lifecourse.

## 1 INTRODUCTION

The global rate of overweight/obesity continues to rise with up to 1.9 billion people impacted, 650 million of whom are obese (Blüher, 2019). While these increases have been well documented in high income countries, increases in low to middle income countries with rapid urbanisation are rising at a more accelerated rate (NCD Risk Factor Collaboration (NCD‐RisC), 2017) further increasing health disparities in these regions. Increasing rates of obesity in child and adolescent populations are also of particular concern as earlier onset of obesity is heavily linked with increased incidence and severity of comorbidities at an earlier age (Woolford et al., 2021). These comorbidities are wide ranging and include type 2 diabetes, cardiovascular disease, musculoskeletal disorders, as well as some forms of cancer. Perhaps less well recognised is the increased risk of infectious disease. The recent COVID‐19 pandemic demonstrated that those living with obesity had increased risk of infection as well as more severe outcomes (Singh et al., 2022). In addition to the significant personal costs, obesity and its comorbidities come with an enormous economic cost (Ling et al., 2023) with a recent report predicting the cost of childhood obesity on the island of Ireland at 7.2 billion euros (Perry et al., 2017). It is therefore not surprising that international organisations such as the World Health Organisation (WHO) have made reduction of overweight/obesity a priority.

While obesity was traditionally considered a simple energy surplus disorder, it is now clear that it is a complex multifactorial disorder. While physiological factors such as genetics and epigenetics interact with environmental exposures such as diet and sedentary behaviour, broader societal factors cannot be ignored. There is a growing body of evidence that highlights cultural influence, food production and availability, rapid urbanisation and stress as additional factors in the onset of obesity. However, one of the strongest factors for predicting obesity is parental obesity and birthweight (Beyerlein et al., 2014). In addition to familial eating behaviours and food environments, it is now clear that early life exposure to environmental stressors such as diet from preconception through to early childhood plays a role in establishing metabolic thresholds that promote excess adiposity in the longer term. In many countries up to two‐thirds of women enter pregnancy either overweight or obese. This increases the risk of pregnancy complications such as gestational diabetes, pre‐eclampsia, pre‐term birth and caesarean section or instrumental delivery. There is also increased risk for miscarriage, still‐birth, neural tube defects and macrosomia. Further, these infants are more likely to become obese themselves and develop cardiometabolic disease. In addition, studies are increasingly demonstrating a role for paternal obesity as a risk factor for offspring obesity. Increased paternal body mass index (BMI) not only reduces reproductive potential but has been shown to increase risk of metabolic complications in the offspring, particularly females (Johnson et al., 2023).

Over four decades ago, British epidemiologist Professor David Barker identified early life as a critical period for mediating the lifelong risk for cardiometabolic disease (Barker et al., 1989). Since then, researchers have identified a range of human cohorts and experimental models across several species, as well as exposures to identify the mechanisms which underly the links between early life adversity and adult disease, in a field of study now coined the Developmental Origins of Health and Disease (DOHaD). Early studies focused on undernutrition/malnutrition as the main driver of this developmental programming, using historical events such as the Dutch Hunger Winter as quasi‐experimental models (Schulz, 2010), along with rodent restriction models that incorporated either low protein or global undernutrition as a maternal stressor (Jazwiec & Sloboda, 2019). These types of diets are less relevant in modern society, therefore recent research has tended to focus on Western diets, which are rich in refined sugars, saturated fats and salt.

These models, both undernutrition and hypercaloric, generally exhibit a common pattern of physiological adaptations including reduced nephron endowment in the kidney, reduced proliferation of muscle fibres and cardiomyocytes and reduced pancreatic β‐cell proliferation, all contributing to the development of cardiometabolic disease. Recent evidence has also demonstrated that early life programming limits adipose tissue capacity (Lecoutre & Breton, 2015). This review aims to explore adipose tissue development and how it is impacted by exposure to parental diet and obesity. We will also review the impact of inflammation on adipose tissue dysfunction and whether early life anti‐inflammatory interventions can negate the effects of adverse early life environments on metabolic health.

## 2 DEVELOPMENT OF ADIPOSE TISSUE

While adipose tissue was traditionally thought of as a relatively inert energy storage depot, it is now considered a dynamic endocrine organ with an essential role in metabolic health, thermoregulation, skin wound healing capacity and reproduction. In addition to fat cells (adipocytes), the adipose tissue contains a diverse range of cell types, including adipocyte precursor cells (pre‐adipocytes), fibroblasts, immune cells, neuronal, vascular and endothelial cells (Ramakrishnan & Boyd, 2018). This non‐adipocyte fraction is referred to as the stromal vascular fraction (SVF). This variety of cells allows the adipose tissue to adapt to different physiological cues ensuring energy distribution in response to the needs of the organism. Additionally, the adipose tissue is arranged into several distinct depots around the body with differing functions and developmental trajectories. These are further divided into three distinct classes, beige, white (WAT) and brown adipose tissue (BAT).

The BAT is located in several depots around the body (cervical, supraclavicular, paraspinal and perirenal regions) and plays a major role in the maintenance of body temperature through a process known as non‐shivering thermogenesis. It is distinguished from the white adipose tissue via multilocular lipid droplets and a high abundance of mitochondria. These mitochondria produce uncoupling protein 1 (UCP1) which dissipates the proton electrochemical gradient across the inner mitochondrial membrane in a process called uncoupling that is responsible for the release of heat. This process utilizes metabolic substrates such as triglycerides, glucose, fatty acids and amino acids (McNeill et al., 2020). Increased BAT abundance is associated with greater metabolic health (Chondronikola et al., 2014), which has led to research focused on therapeutic strategies to increase BAT abundance and activation. Beige adipocytes are similar to brown adipocytes in that they have multilocular lipid droplets and increased numbers of mitochondria. However, they do not possess distinct depots and are found within the WAT. They form from a distinct subset of pre‐adipocytes or via transdifferentiation in the WAT and are activated in response to a number of environmental stimuli including diet, exercise and pharmaceutical agents and are thought to confer beneficial effects in relation to metabolic health (Pilkington et al., 2021).

For the most part, the remaining sections of this review will examine the WAT. While exceptions occur, most species store fat in the WAT. Developmentally, the WAT originates mainly from the mesoderm via sequential proliferation and differentiation of adipoblast cells to preadipocytes (Gesta et al., 2007). The development of mature adipocytes is termed adipogenesis and is reliant on four distinct stages, growth arrest, clonal expansion, early differentiation and terminal differentiation. The transcription factors peroxisome proliferator activated receptor γ (PPARγ) and CCAAT/enhancer‐binding‐protein‐α (C/EBPα) are major regulators of differentiation and maintenance of terminal maturation during adipogenesis (Rosen & MacDougald, 2006). Recent studies have demonstrated that dysregulation of these transcription factors occurs in offspring from obese mothers, resulting in increased preadipocyte proliferation at birth (Sousa & Rodrigues, 2023). Indeed, cord derived‐mesenchymal stem cells from infants whose mothers were obese displayed alteration in adipogenesis and adipocyte hypertrophy (Keleher et al., 2023) and can predict adiposity in 4‐ to 6‐year‐old children. In rodents most adipose tissue development occurs in the postnatal period whereas in humans WAT development occurs early in the second trimester and it is relatively well developed by birth. However, recent lineage tracing studies have identified the late fetal period as a critical window for the development of the adipocyte progenitor pool. Environmental cues such as maternal nutrition that impede the development of this progenitor pool can have significant implications for adipogenic capacity over the life course. Within the WAT there are several distinct depots, the visceral adipose tissue (VAT), which is found around central organs such as the liver, kidneys and gut, and the subcutaneous adipose tissue (SAT), which is found under the skin. Both make individual contributions to metabolic health and display significant differences in histological structure and molecular signatures. It is thought that differential developmental trajectories are responsible for these differences (Gesta et al., 2007).

Further, these tissues tend to be responsive to different stimuli, with adipose tissue in the breasts and thighs responsive to sex hormones such as oestrogen, while adipose tissue in the neck and back is responsive to glucocorticoids. This induces a sex‐specific pattern of adipose tissue distribution with women more likely to store adipose tissue in the gluteofemoral regions, while men preferentially store fat in visceral depots (Karastergiou et al., 2012). Indeed, oestrogens tend to be protective against metabolic disease, which may be due to its influence on the adipose tissue, with effects including reduced adipocyte size, lipogenesis, lipolysis and increased mitochondrial activity (Davis et al., 2013; Nookaew et al., 2013). While it is tempting to speculate that these sex‐specific differences are solely due to sex hormones, it is clear that in utero environments can induce sex‐specific differences on long term adipose tissue function. It is thought that genes affecting adiposity are present on the X chromosome and gene dosage effects in the fetus may influence adiposity in addition to oestrogen. Another potential mechanism is the placenta, a fetal organ that is known to display sex‐specific effects in relation to size, efficiency and nutrient transport. It is thought that male placentas are less adaptable to environmental changes due to lower expression of an X‐linked gene, *O‐GlcNAc*, whose product mediates epigenetic processes (Dearden et al., 2018).

The Dutch Hunger Winter represents an example of how the in utero environment can influence sex‐specific effects in the adipose tissue. Exposure to famine in early or late gestation resulted in increased adiposity and dysregulated lipid profiles in female but not male offspring (Lumey et al., 2009; Ravelli et al., 1999). Studies focusing on maternal obesity have also shown dysregulated adipose tissue in the offspring, with increased adiposity in male children by 6 years of age (Andres et al., 2015) and altered growth patterns in female rather than male children from 0 to 7 years of age, in response to maternal pre‐pregnancy obesity (Oostvogels et al., 2017). The effects of maternal diet‐induced obesity have also been extensively examined in animal models, with increased adiposity evident in offspring from high‐fat or high‐sugar fed mothers (Dearden & Balthasar, 2014). Further work by Smith et al. (2022) has shown that early‐life fructose exposure programmes metabolic dysfunction in offspring by initiating *de novo* lipogenesis and mitochondrial metabolism in a guinea pig model. It is clear that the mechanisms that influence adiposity early in life are complex, with maternal nutritional intakes representing a significant factor. However, the role of the father cannot be discounted.

Work over the last decade has demonstrate that paternal factors such as diet, BMI and age can influence the growth and development of the fetus. Indeed, Zhang et al. (2023) demonstrated that paternal obesity influences in vitro fertilisation outcomes with a reduced likelihood of generating high quality embryos and increased risk for macrosomia and large for gestational age in neonates. Another recent study examined the impact of paternal BMI on maternal and offspring outcomes in a large Chinese cohort. They demonstrated that there were increases in maternal complications such as gestational weight gain, hypertension and C‐section when fathers were overweight or obese (Lin et al., 2023). There was also evidence that by adolescence, offspring were at increased risk for obesity and asthma. They carried out a subgroup analysis to determine if these effects were influenced by maternal BMI, and while this had a significant effect, paternal factors maintained their significance. Johnson et al. (2023) demonstrated in a pooled analysis of UK cohorts from a 1958 child development survey and the 2001 Millenium cohort studies that parental obesity was strongly associated with overweight and obesity at 7 and 17 years of age. They also identified that childhood BMI rates have increased fourfold.

While the impact of paternal BMI is clear in human studies, less is known in relation to the contribution of specific nutrients or dietary patterns. However, rodent models utilising high fat models of paternal obesity have been useful in determining the effects of nutrition and understanding the mechanisms which may contribute to paternal obesity‐induced programming of offspring outcomes. Initial work by Morris and colleagues demonstrated that paternal high fat diets (HFD) program pancreatic β‐cell dysfunction in the absence of increased adiposity but only in female Sprague–Dawley rat offspring (Ng et al., 2010). Despite no change in weight, these offspring had significant transcriptomic changes in pathways involved in olfactory genes, oxidative stress and mitochondrial function in the retroperitoneal adipose tissue (Ng et al., 2014). Male offspring from HFD‐fed fathers were smaller at birth and had reduced weight and adipose tissue mass by 6 months of age. This was associated with a downregulation of gene expression in adipogenic pathways (Lecomte et al., 2017). While there is little evidence of nutritional interventions in this area, a study by Krout et al. (2018) demonstrated that paternal exercise protects offspring from HFD‐induced metabolic dysfunction via increased PPARγ‐induced beigeing of WAT.

## 3 INFLAMMATION AND ADIPOSE TISSUE DYSFUNCTION

Inflammation is an important process which is instrumental in mediating tissue repair and defence against harmful pathogens such as viruses and bacteria. However, when this process runs unchecked, it can be harmful and contribute to tissue damage and progression of conditions such as inflammatory bowel disease (IBD), insulin resistance and arthritis. Indeed, obesity and cardiometabolic disease have been associated with chronic low‐grade inflammation. Adipocyte precursor cells within the adipose tissue produce chemoattractant proteins called chemokines (macrophage chemoattractant protein (MCP)‐1 and C‐C motif receptor (CCR)‐2) which attract immune cells, including macrophages (Kaplan et al., 2015). These macrophages drive inflammation leading to disruption of normal biological processes such as insulin signalling and lipid synthesis in the adipose tissue, resulting in increased circulating free fatty acids (FFA) and heightened glucose concentrations and subsequently impacting other organ systems (Lee & Lee, 2014). This includes the liver, where increased FFA can increase the risk of non‐alcoholic fatty liver disease (Qureshi & Abrams, 2007) and the pancreas where increased glucose concentrations put excess stress on the pancreatic β‐cells resulting in the development of type 2 diabetes (Gerst et al., 2019). While these relationships are well teased out, there are data suggesting that increasing BMI and adipose tissue inflammation can have far ranging effects, impacting the skin (acne severity and poor wound healing) (Melnik, 2016) and even predisposing to certain types of cancers (Deng et al., 2016).

Macrophages represent an important mediator of these processes. They are a type of white blood cell that are present in almost all tissues and are critical for maintaining tissue homeostasis. They are highly plastic cells which are epigenetically programmed by their microenvironment and as such are involved in a wide range of functions from injury and infection response to tissue repair. Traditionally, macrophages have been grouped into two broad classifications, the ‘M1’ pro‐inflammatory macrophage, which produces pro‐inflammatory cytokines such as interleukin (IL)‐1β and tumour necrosis factor (TNF)‐α, and ‘M2’ anti‐inflammatory/wound resolving macrophages, which instigate wound repair and produce cytokines such as IL‐10, IL‐4 and transforming growth factor (TGF)‐β. In a lean state, macrophages make up a majority of the non‐adipocyte fraction of the adipose tissue (Weisberg et al., 2003). These macrophages are typically in an M2 state and serve to regulate insulin sensitivity, tissue repair and remodelling. In an obese state, polarisation to a pro‐inflammatory M1 state occurs and the resulting cytokine production inhibits insulin signalling pathways resulting in metabolic dysfunction (Chakarov et al., 2022). However, recent advances in this area have shown that the classification of macrophages into M1/M2 phenotypes is an oversimplification and a broad range of subtypes exist. Indeed, Kratz et al. (2014) have recently identified a ‘metabolic activation’ macrophage phenotype which is based on the obese adipose tissue microenvironment. This is a subset of macrophages which is stimulated with FFA, insulin and glucose and is distinct from M1 macrophages in that it is reliant on expression of PPARγ, a marker typically associated with M2 phenotypes. As obesity and its associated inflammatory state is a global condition, affecting multiple organ systems, it is important to understand the role and function of these macrophage subtypes in order to maximise treatment strategies. There have been several approaches designed to reduce this metabolic inflammation, including nutritional strategies, exercise interventions and overall lifestyle interventions. Exercise interventions are reviewed in Harris et al. (2018), but the main focus of this review is dietary interventions.

Studies utilising anti‐inflammatory nutrients have demonstrated a reduction in pro‐inflammatory cytokines in adipose tissue and subsequent beneficial effects in terms of overall metabolic health, underscoring the importance of nutrition in the processes described above. Indeed, diet is one of the strongest environmental regulators of inflammation. Fats are the most comprehensively explored macronutrient in relation to obesity‐induced inflammation. Work over the last 20 years has shown that saturated fats initiate immune reactions that result in the low‐grade chronic inflammation that is associated with obesity. They do this through activation of the toll‐like receptor (TLR)–IL‐1 and the NLRP3 inflammasome signalling pathways, which promotes release of pro‐inflammatory cytokines (Huang et al., 2012; McGillicuddy et al., 2011; Reynolds et al., 2012). These pathways then disrupt insulin signalling pathways promoting metabolic dysfunction and eventual type 2 diabetes. However, not all fats have this detrimental phenotype; mono‐ and omega‐3 long‐chain poly‐unsaturated fats (MUFA and *n*−3 PUFA, respectively) are anti‐inflammatory in nature and promote resolution of inflammation, providing a beneficial impact on metabolic health (Finucane et al., 2015; Noureddine et al., 2022). This occurs through downregulation of TLR4/IL‐1 signalling and increased expression of PPARγ, which promotes polarisation of macrophages to an anti‐inflammatory phenotype. Indeed, preclinical studies have demonstrated that dietary substitution of mono‐unsaturated fats for saturated fat has beneficial effects on insulin concentrations and inflammation, resulting in an overall benefit to metabolic health (Ralston et al., 2020). Indeed, lipid mediators are not the only anti‐inflammatory compounds which can impact metabolic inflammation: polyphenols also represent promising targets given their innate antioxidant properties. While these have been well investigated within the context of obesity and metabolic dysregulation, much less is known in relation to their potential impacts during pregnancy complicated with obesity and developmental programming of offspring adipose tissue dysfunction. It is clear, however, that resolution of adipose tissue inflammation has promising effects in terms of metabolic dysfunction. Therefore, it is tempting to speculate that anti‐inflammatory nutritional interventions during pregnancy can improve outcomes in the offspring.

## 4 DIETARY INTERVENTIONS

Table 1 summarises dietary anti‐inflammatory dietary interventions in mothers and their impact on offspring adipose tissue.

**TABLE 1:** Summary of dietary anti‐inflammatory interventions.

Intervention	Species	Author/study	Outcomes
Fish oil	Human RCT	Brei et al. (2016), Hauner et al. (2012), Much et al. (2013) (INFAT, Germany)	No significant difference in fat mass; positive association of maternal DHA and *n*−3 LC‐PUFA with birth weight; sexually dimorphic placental miRNA
		Haghiac et al. (2015) (USA)	Decreased IL‐6, IL‐8, TNF‐α and TLR4 in adipose/placenta; 17% reduction in fasting triglyceride; lower rate of caesarean section delivery
		Satokar et al. (2022) (New Zealand)	
Fish oil	Rodent models	Sardinha et al.	Lower body weight in male offspring; reduced insulin concentrations; increased glucose tolerance
		Casas‐Agustench et al.	Different fat sources altered AT miRNA in mothers
		Hussain et al. (beef vs. herring‐based diets)	Herring fed offspring had reduced body weight and greater insulin sensitivity
Fish oil + HFD		Satokar et al.	Increased insulin sensitivity in both male and female offspring (HFD only)
		Ramalingam et al.	Increased insulin sensitivity in both male and female offspring; increased adipokine concentrations
CLA + HFD	Rodent models	Segovia et al. (2015; 2017), Reynolds et al. (2015) (Sprague–Dawley rat model)	Prevented HFD‐induced fetal weight reduction; in male offspring at weaning reduced body weight, AT weight and insulin concentrations; reduced adult offspring (male but not female) body weight, adipocyte size, adipogenic gene expression; prevented increased circulating lipids, hepatic lipogenic gene expression and oestrus irregularities in female offspring from HF fed mothers.
		Gonzalez et al. (Wistar rat model)	CLA reduced HFD induced body weight and fat mass, normalised TAG concentrations
		Lavandera et al. (2017)	Programming effect on lipid metabolism pathways; prevented TAG accretion in AT and liver
Resveratrol + HFD	Rodent models	Ros et al. (2018, 2020)	Prevented increased body weight, VAT and SAT in offspring from HFD‐fed mothers; fasting glucose increased in both sexes; altered AT gene expression differently based on diet and sex.
		Tsai et al. (2020) (male offspring only)	Reduced body weight, insulin, TAG, fat mass in mothers and male offspring; induced BAT‐like changes in adipose tissue; improved glucose tolerance
		Zou et al., 2017	Resveratrol prevented HFD‐induced increases in body weight, insulin, triglycerides, fat mass in mothers and male offspring; induced BAT‐like changes in WAT; improved glucose tolerance in male offspring
Curcumin	Rodent model	Santos et al. (2023)	Improved cholesterol, glucose and TAG concentrations in mothers and male offspring
	Human *ex vivo* study	Nguyen‐Ngo et al. (2020)	Suppressed inflammation in maternal AT collected from women during C‐section
EGCG + HFD	Rodent models	Li et al. (2012) (Sprague–Dawley)	Prevented hyperinsulinaemia associated with HFD; increased insulin sensitising markers in AT of male offspring
		Hachul et al. (2018a, 2018b)	In mothers, increased adiponectin, TNF‐α and IL‐1β in AT. Lower fat mass but increased cytokines; in offspring, improved glucose tolerance

AT, adipose tissue; CLA, conjugated linoleic acid; DHA, docosahexaenoic acid; EGCG, epigallocatechin gallate; HFD, high‐fat diet; LC‐PUFA, long‐chain polyunsaturated fatty acids; RCT, randomized controlled trial; SAT, subcutaneous adipose tissue; TAG, triacylglycerol; VAT, visceral adipose tissue; WAT, white adipose tissue.

### 4.1 Anti‐inflammatory lipids

#### 4.1.1 Fish oil/eicosapentaenoic acid/docosahexaenoic acid

Fish oil supplementation is one of the most extensively studied anti‐inflammatory interventions during pregnancy, with several experimental rodent models as well as human randomized controlled trials (RCTs) carried out to date. Fish oil is rich in the omega‐3 long‐chain polyunsaturated fatty acids (LC‐PUFA) eicosapentaenoic acid and docosahexaenoic acid (DHA). These fatty acids have anti‐inflammatory properties that have potential beneficial effects across a range of inflammatory conditions such as Crohn's disease (Cabré et al., 2012), arthritis (Raad et al., 2021) and cardiometabolic disease (Vors et al., 2021). However, results from clinical studies can be contradictory with some showing no impact on disease symptoms. in vitro analysis demonstrates that fish oil treatment can reduce expression of genes involved in adipogenesis, lipogenesis and β‐oxidation of fatty acids (Isesele et al., 2022). Further, there is evidence that increased maternal *n*−3 PUFA concentrations in the plasma have impacts on SAT in infants (Jelena Vidakovic et al., 2016). These effects along with evidence that *n*−3 LC‐PUFA are important for the developing fetus make it a promising target for preventing diet‐induced developmental programming of obesity.

#### 4.1.2 Human studies

A number of RCTs have examined the effects of fish oil supplementation during pregnancy. The INFAT study based in Germany examined the impact of dietary modification of the *n*−6:*n*−3 LC‐PUFA ratio in pregnant and lactating women on infant and childhood adipose tissue growth (Brei et al., 2016; Hauner et al., 2012). This intervention in healthy women (*n* = 208) involved supplementation with fish oil together with dietary counselling aimed at lowering arachidonic acid intake, while the control group received general recommendations in relation to healthy diet. Adiposity measurements were conducted at 2, 3, 4 and 5 years of age using ultrasound to determine abdominal, SAT and preperitoneal fat depots with magnetic resonance imaging analysis conducted on a subset of 5‐year‐old children. Neither study observed any significant differences in fat mass between the two groups; however, there was a significant difference in weight and BMI between groups in unadjusted statistical models at 4 years of age. Despite no changes between groups, it was found that maternal DHA and *n*−3 LC‐PUFA were positively associated with birth weight and maternal red blood cell arachidonic acid and *n*−6 PUFA negatively associated with BMI and ponderal index at 1 year (Much et al., 2013). A further follow‐up study examined miRNA targets in the placenta and cord blood and found sexually dimorphic molecular regulation in the placenta (Sedlmeier et al., 2021).

Fish oil supplementation has also been examined within the context of overweight/obese pregnancy in both a US‐based cohort (2 g fish oil; *n* = 36) and a New Zealand cohort (6 g of fish oil) using a double‐blind RCT approach (Haghiac et al., 2015; Satokar et al., 2023). Neither of these studies resulted in changes in maternal insulin resistance. However, beneficial effects were seen in both studies. Haghiac et al. showed that women receiving fish oil supplementation had decreased expression of IL‐6, IL‐8, TNF‐α and TLR4 in adipose and placental tissues, while Satokar et al. demonstrated that treatment throughout pregnancy and up to 3 months’ lactation (where women were still breastfeeding their infants) resulted in a 17% reduction in fasting triglyceride concentrations. This appears to be the only study which examined the oxidation status of the fish oil used in the intervention. This appears to be an important consideration, as previous work by this group using a rat model demonstrated that exposure to oxidised fish oil during pregnancy increased neonatal mortality, altered placental genes relevant to antioxidant pathways and increased the production of free radicals (Satokar et al., 2022). Contrary to Much et al. (2013) there were no differences in infant size at birth. Percentage body fat at 2 weeks and 3 months was determined using dual‐energy X‐ray absorptiometry analysis, and while there were no differences between groups, infants whose mothers were supplemented with fish oil had a greater ponderal index and BMI *z*‐score at 3 months of age. There was a sex‐specific effect at 3 months with female infants where fish oil supplementation was associated with increased body fat percentage, weight, ponderal index and BMI *z*‐score. This increase in fat mass was attributed to greater peripheral rather than central fat mass.

The importance of *n*−3 PUFA concentrations in human milk has also been demonstrated and is a potentially important factor in early infant adipose tissue deposition independent of maternal BMI. Rudolph et al. (2017) examined 48 infants from normal weight, overweight and obese women who had exclusively breastfed. They found that women living with obesity had lower concentrations of DHA and higher *n*−6/*n*−3 PUFA ratios in their milk at both 2 weeks’ and 4 months’ lactation. Using air displacement plethysmography, they assessed infant body composition and found that infant fat mass was predicted by *n*−6/*n*−3 PUFA ratios. This shows that the impact of early life nutritional environment is not just restricted to pregnancy and that lactation is a critical period for determining the way in which infant adipose tissue develops.

#### 4.1.3 Animal studies

A number of rodent models have also examined the impact of maternal fish oil supplementation on offspring adipose tissue outcomes. Sardinha et al. (2013) examined the impact of fat source (including fish oil) during the first 12 days pregnancy on offspring outcomes. At 8–12 months of age male but not female offspring from fish oil‐fed mothers had lower body weights and insulin concentrations accompanied by increased glucose tolerance than offspring exposed to olive oil, linoleic acid and palmitic acid. Casas‐Agustench et al. (2015) further examined the impacts of these diets on adipose tissue miRNA content in mothers and offspring. They observed that different fatty acid sources can modulate miRNA profiles in a differential manner in peripheral maternal tissues, which may have implications for intergenerational programming of offspring metabolic health. Similar results were seen in C57BL/6 mice fed fish oil from whole food sources (herring‐based vs. beef‐based) (Hussain et al., 2013). Given the food matrix effect and the additional nutrient content derived from whole food sources these diets may be more applicable to a human situation.

Rudolph et al. (2018) demonstrated that manipulation of genes involved in LC‐PUFA processing can prevent the negative impacts of a maternal HFD. They utilised a mouse model that overexpressed an *n*−3 fatty acid desaturase (fat‐1), thereby lowering the *n*−6/*n*−3 PUFA ratio and found that maternal fat‐1 transgene expression reduced the *n*−6/*n*−3 PUFA ratio in milk and that this carried through to the plasma fatty acid composition of their offspring. These offspring had smaller adipocyte size but greater adipose tissue mass by postnatal day 14, which was accompanied by increased adiponectin concentrations. Further molecular analysis demonstrated that transgenic animals had downregulation of genes involved in lipid metabolism and adipogenesis. This appears to be driven by increased methylation and subsequent reduced expression of PPARγ in the SAT. A separate cohort was brought to adulthood and exposed to a high fat, high sugar diet. *Fat1* transgenic offspring had significantly reduced body fat mass compared to controls accompanied by lower energy balance, fasting glucose and improved glucose tolerance in response to an oral glucose tolerance test.

Fish oil supplementation was also examined in combination with a HFD (Ramalingam et al., 2021; Satokar et al., 2022). In both studies, maternal supplementation with fish oil resulted in increased insulin sensitivity in both male and female offspring compared to HFD alone. While Ramalingam et al. showed additional beneficial effects such as increased adipokine concentrations and reduced gene expression of *Mcp1* and *Tnfa* (female only), both groups demonstrated increased gonadal fat mass/body weight. Furthermore, Satokar et al. showed fish oil supplementation in a low‐fat setting resulted in impaired insulin sensitivity, increased body weight and reduced cardiac ejection fraction in male and female offspring. Key differences between these studies include the lack of a control diet fish oil group, and therefore any potentially adverse effects of fish oil intake during healthy pregnancy were not captured, and the administration of the fish oil, which Ramalingam incorporated into the food (potentially risking oxidation) but Satokar provided in jelly that was rapidly consumed.

These results suggests that fish oil supplementation may not be a one size fits all strategy, and treatment regimes should be targeted to women who are suspected of having metabolic issues.

#### 4.1.4 Conjugated linoleic acid

Conjugated linoleic acid (CLA) refers to geometric and positional isomers of linoleic acid, of which there are 28. The main route of synthesis comes from the biohydrogenation of linoleic acid during rumen fermentation and synthesis in the mammary gland of ruminants through desaturation of *trans*‐vaccenic acid. Ruminant diet is a main factor in the production of CLA, with pasture rather than grain fed animals yielding higher concentrations. The main dietary sources of CLA are dairy and ruminant meat products. *c*9,*t*11‐CLA and *t*10,*c*12‐CLA isomers are the most abundant and are generally found in a 10:1 ratio (Shen & McIntosh, 2016). CLA has potent inflammatory actions which are exhibited in an isomer specific manner. *c*9,*t*11‐CLA is an agonist of PPARγ and has shown benefit in a range of pro‐inflammatory conditions such as arthritis (Aryaeian et al., 2014; Butz et al., 2007), colitis (Bassaganya‐Riera & Hontecillas, 2010) and cardiometabolic disease (Kim et al., 2016; Reynolds & Roche, 2010). The anti‐obesogenic effects of CLA are typically attributed to the *t*10,*c*12‐CLA rather than the *c*9,*t*11‐CLA isomer. This isomer reduces adipogenesis and inflammation while increasing lipolysis and fatty acid oxidation (Kennedy et al., 2010). These effects are not without controversy, however, as studies have shown that while CLA can reduce BMI by initiating weight loss, longer term supplementation can reduce high‐density lipoprotein cholesterol (Kim et al., 2016). CLA potentially represents an attractive nutraceutical option for preventing adipose tissue inflammation and subsequent dysfunction, and while this has been explored in adult feeding studies, there is limited information in regard to the impact of CLA supplementation during pregnancy and lactation on maternal and offspring adipose tissue function and metabolic health. Given that CLA crosses the placenta, it has the potential to influence fetal developmental processes (Murru et al., 2022).

There are several animal models examining the impact of CLA supplementation during pregnancy on offspring adipose tissue. Rodent models in this area which incorporate both obesogenic diets and CLA supplementation are important in deciphering potential mechanisms which might be relevant to human health.

Segovia et al. (2015) present data examining the impact of 1% CLA supplementation alone and in combination with a HFD during pregnancy and lactation in Sprague–Dawley rats. They found that CLA supplementation both alone and with a HFD increased maternal body weight to the same extent as HFD alone. Despite this, CLA in both control and HFD settings reduced fasting plasma lipids (triacylglycerol, low‐density lipoprotein cholesterol) and cytokines (IL‐1β, TNF‐α, leptin) compared to HFD alone, but no difference was seen in glucose or insulin concentrations. The reduced fetal weight (both male and female) observed with HFD was prevented when mothers were supplemented with CLA. By weaning, offspring from CLA‐supplemented mothers had reduced body weight, retroperitoneal adipose tissue weight and insulin concentrations (male only) as well as increased indexes of insulin sensitivity. A follow‐up paper from this study examined the impact of CLA supplementation in the setting of maternal obesity in adult offspring (Segovia et al., 2017). It was found that maternal CLA supplementation reduced HFD‐induced increases in body weight and fat mass in offspring. An oral glucose tolerance test showed that adult male (but not female) offspring from obese mothers supplemented with CLA had enhanced glucose and insulin sensitivity compared to those whose mothers were not supplemented with CLA. This was accompanied by a reduction in adipocyte size and reduced *Pref‐1* gene expression in the adipose tissue. While there was no change in *Pparγ* gene expression, they speculated that reduced Pref‐1 prevented maturation of new adipocytes limiting the adipogenic capacity of offspring from obese mothers, something that was prevented with maternal CLA supplementation. While these effects were only observed in male offspring, female offspring from HFD‐fed mothers displayed increased circulating lipids and hepatic lipogenic gene expression compared to female offspring of mothers supplemented with CLA (Reynolds et al., 2015).

González et al. (2020) presented a similar study examining the effects of maternal CLA supplementation in response to a HFD in Wistar rats, the main difference from Segovia et al. (2017) being lack of a CLA supplementation on a control background and data only presented for male offspring. They also maintained their offspring on the maternal diet background after weaning and incorporated an extra postnatal diet in the maternal HFCLA group so that offspring received either a HFD or HFCLA. The study design was relatively unbalanced making it difficult to disentangle pre‐ and post‐natal impacts of CLA supplementation. Nonetheless, they found that HFD plus CLA supplementation in both pre‐ and post‐natal diets reduced body weight and fat mass. They found that HFD increased triacylglycerol concentrations through a reduction of lipogenic genes; this was normalised with CLA supplementation. Lavandera et al. (2017) examined maternal CLA supplementation in the absence of an obesogenic background in Wistar rats. After weaning, male offspring were separated into either control or CLA diet for the remainder of the study. They found that CLA had a programming effect on lipid metabolism pathways resulting in a prevention of TAG accretion in the adipose tissue and liver; these metabolic effects were attributed to the maternal dietary exposure rather than adult exposure to CLA.

### 4.2 Polyphenols

#### 4.2.1 Resveratrol

Resveratrol is a stilbenoid polyphenol that is found in the skin of grapes, berries and peanuts. It displays anti‐oxidant, anti‐inflammatory and cardioprotective effects and has been shown to be beneficial in inflammatory conditions by reducing cyclooxygenase (COX)1 and COX2 activity and inhibiting nuclear factor κB (NF‐κB), a transcription factor that initiates inflammatory cascades, and subsequent cytokine production (Zhou et al., 2018). However, there is evidence that it is effective only in high doses due to limited bioavailability. This is mainly due to its poor water solubility as well as its rapid metabolism and elimination (Walle, 2011). While the effects of resveratrol have been relatively well characterised in relation to obesity and cardiometabolic disease (Salehi et al., 2018; Walle, 2011), its effects during pregnancy and offspring health are less well known in humans. Its anti‐obesity and protective cardiometabolic effects coupled with its ability to cross the placenta (Bourque et al., 2012) make it an attractive target for preventing developmental programming. Indeed, several rodent studies have examined the effectiveness of resveratrol intake during pregnancy on offspring adipose tissue function.

Ros et al. (2018) adopted a balanced factorial design in Wistar rats which involved control and HFD during pregnancy and lactation supplemented with either normal drinking water or 50 mg resveratrol. By weaning, male and female pups from HFD‐fed mothers had increased VAT and SAT mass. Exposure to resveratrol in utero prevented this gain in body weight, VAT (females only) and SAT. Fasting glucose was increased by resveratrol in both sexes but there were no changes in insulin, homeostatic model assessment for insulin resistance, plasma lipids, or adipokines in response to resveratrol. Maternal HFD increased gene expression of markers involved in lipogenic pathways and fatty acid uptake, but this was unaffected by resveratrol.

Resveratrol reduced body weight in offspring from HFD‐fed mothers but increased it in control diet‐fed mothers (Ros et al., 2020). The effect of maternal resveratrol intake on *Pparγ* expression in VAT depended on the maternal diet; resveratrol increased *Pparγ* mRNA expression in rats from HFD mothers but decreased it in offspring from low fat diet dams. Furthermore, resveratrol shifted the distribution of adipocyte size in the adult offspring to a significantly higher incidence of large adipocytes, and this was regardless of sex or maternal diet. While similar results were found by Tsai et al. (2020), they also observed that resveratrol reduced body weight and adipocyte size in offspring irrespective of diet. Gene expression analysis in the adipose tissue showed that maternal resveratrol supplementation reduced expression of *Acl* and *Acc2*, indicating alteration in lipogenic pathways. This study was only carried out in male offspring, limiting the applicability of the results.

Zou et al. (2017) examined whether maternal resveratrol supplementation could facilitate beige adipogenesis of WAT and thus improve metabolic health in C57/Bl6 mice. They incorporated resveratrol into either control diet or HFD allowing them to examine the impacts of maternal HFD, maternal resveratrol exposure and whether an interaction existed between these groups. However, only male pups were used for this study, limiting applicability and translatability. In mothers, resveratrol prevented HFD‐induced increases in body weight, insulin, triglycerides and fat mass. Similar effects were seen in male offspring at weaning. Additionally, they demonstrated that maternal resveratrol supplementation increased BAT metabolism and induced BAT‐like changes in WAT in male HFD offspring. They found that by adulthood, male offspring from resveratrol supplemental HFD‐fed mothers had reduced adipose tissue mass and increased glucose tolerance compared to HFD alone.

Despite promising results in animal models there is little evidence in relation to the impact of resveratrol in humans. However, Tran et al. (2017) examined omental adipose tissue explants from humans without pregnancy complications, obtained during caesarean section. These explants were included with 200 µM resveratrol and stimulated with an inflammatory stimulus, TNF‐α, IL‐1β or lipopolysaccharide. Resveratrol treatment reduced gene expression and medium concentrations of key cytokines including IL‐1β, IL‐1α, IL‐6 and MCP‐1. While no information from offspring was presented, resveratrol has clear anti‐inflammatory potential in the adipose tissue, which may represent a mechanism for improving both maternal and offspring health.

#### 4.2.2 Curcumin

Resveratrol is not the only polyphenol that has been examined in this context. Curcumin is the main natural polyphenol found in the rhizome of turmeric. It exhibits anti‐inflammatory potential by regulating pro‐inflammatory signalling mediators such as NF‐κB, mitogen‐activated protein kinases via activation of PPARγ (Peng et al., 2021). These anti‐inflammatory effects have proven beneficial in a range of inflammatory conditions including arthritis (Atabaki et al., 2020), Crohn's disease (Sugimoto et al., 2020) and metabolic syndrome (Panahi et al., 2016). Less is known in relation to its role in adipose tissue, but several in vitro studies have shown promotion of beige adipogenesis and suppression of adipocyte differentiation (Jin et al., 2018) making it a potential candidate for impacting adipose tissue development in utero.

Santos et al. (2023) orally gavaged Swiss mice exposed to a hyperglycaemic diet with curcumin during pregnancy and lactation. However, the supplementation regime was only implemented in mice receiving the hyperglycaemic diet, and it was not possible to determine the impact of curcumin on a standard diet. In the post‐natal arm of this study, only males receiving a hyperglycaemic diet were used. This makes it difficult to tease out the impact of maternal programming in isolation, and whether there are sex‐specific effects remains unknown. However, there were clear effects of curcumin supplementation in mothers. While weight or adiposity did not change, there were beneficial effects on biochemical parameters such as cholesterol, glucose and triglyceride concentrations. In male offspring curcumin exposure in utero improved glucose tolerance and insulin concentrations relative to the hyperglycaemic group and promoted increased expression of markers related to thermogenesis in the adipose tissue suggestive of beige adipogenesis.

While postnatal effects were not examined, a study by Nguyen‐Ngo et al. (2020) demonstrated that the polyphenol compounds curcumin and punicalagin suppress inflammation in maternal adipose tissue from normoglycaemic non‐obese women undergoing C‐section. Blunt dissection of adipose tissue was carried out, followed by 20 h incubation with the polyphenol compounds with or without an inflammatory stimulus (TNF). Punicalagin reduced expression of *IL1A* mRNA in VAT and SAT but increased *IL1B* in SAT. Curcumin did not have any impact on expression of cytokines in the VAT but reduced *IL1A*, *IL1B* and *IL6* expression in SAT. The effects of polyphenols were much more pronounced in relation to chemokine expression with both curcumin and punicalagin reducing TNF‐mediated *CCL4, CXCL5, CXCL1* and *CSCL8* expression in VAT. They examined extracellular signal‐regulated kinase signalling pathway as a potential mediator of these effects but found no effect with either punicalagin or curcumin and speculated that the effects may be mediated through other inflammatory signalling pathways such as NF‐κB.

#### 4.2.3 Epigallocatechin gallate

Epigallocatechin gallate (EGCG) is a bioactive flavonoid found in green tea. It has shown potential as an anti‐obesity agent with several in vitro studies demonstrating inhibition of pre‐adipocyte differentiation and lipogenesis while promoting adipocyte apoptosis and fatty acid β‐oxidation. It is thought to act via activation of AMP‐activated protein kinase signalling pathways (Li et al., 2018; Hwang et al., 2005). Three studies have examined the impact of EGCG supplementation in maternal obesogenic diets on offspring adipose tissue function.

Li et al. (2012) examined supplementation of low‐ and high‐fat diets with EGCG during pregnancy and lactation in Sprague–Dawley rats. Similar to other programming studies, the design on this experiment only allowed for analysis of male offspring, and EGCG exposure was only examined in combination with a HFD. Therefore, EGCG exposure in healthy controls and females was not accounted for. In both the dams and their male offspring, EGCG prevented hyperinsulinaemia associated with HFD exposure at both doses. EGCG increased expression of key insulin sensitising markers such as adiponectin, insulin receptor and *Ppargc1* in the liver of mothers and adiponectin, *Glut4* and *Ucp1* the adipose tissue of male offspring, suggesting a beige adipocyte phenotype.

A further study by Hachul et al. (2018a) examined green tea extract versus water in Wistar rats during pregnancy and lactation. Pups and mothers were examined at weaning, but it is not clear whether males or females or a pooled sample was analysed. Green tea extract did not impact body or tissue weights in either pups or mothers, but adiponectin concentrations were increased in the pups. Mothers that were exposed to the green tea extract had increased IL‐10/TNF‐α ratios and IL‐1β in the adipose tissue. The pups had a lower retroperitoneal adipose tissue mass and increases in several adipose tissue cytokines including IL‐10, TNF‐α, IL‐1β and IL‐6. This study shows that green tea extract had a pro‐inflammatory effect contrary to other studies on polyphenols. However, many of the other studies failed to include standard diet controls that included polyphenol supplementation, indicating that there may potentially be negative implications for supplementation outside of a HFD setting.

A follow up study was carried out by Hachul et al. (2018b) that examined adult offspring from control or green tea extract exposed mothers who had been weaned onto either a control or HFD. Only male offspring were utilised. They found that offspring whose mothers were supplemented with green tea extract had improved glucose tolerance compared to control offspring when exposed to a HFD postnatally. While postnatal HFD increased adipose tissue mass in several depots, this was not changed by maternal green tea supplementation. Gonadal adipose tissue cytokine concentrations of IL‐10, IL‐6 and TNF‐α were decreased in the male offspring of mothers supplemented with green tea and exposed to a HFD postnatally. However, offspring from green tea fed mothers exposed to a control diet postnatally had increased concentrations of these cytokines. Western blot analysis showed that this was due to modification of phosphorylated NF‐κBp50.

## 5 CONCLUSION

There is now ample evidence to show that the early life nutritional environment, be it pre‐conception, in utero or the neonatal/lactation period, plays a major role in shaping the adipose tissue. Adverse environments limit the adipogenic capacity of the adipose tissue, creating an environment that heavily influences later risk for cardiometabolic disease. Given the contribution of inflammation to the pathogenesis of conditions such as insulin resistance, type 2 diabetes and heart disease, it is reasonable to assume that reducing this inflammatory load could potentially provide health benefits in the long term. However, as inflammatory processes are critically important for healthy pregnancy processes such as implantation, immune tolerance to the fetus and the induction of labour, the manner in which anti‐inflammatory strategies are administered must be considered. For example, an animal study which examined the impact of ablation of IL‐1 receptor 1 (IL‐1R1), in conjunction with diet‐induced obesity during pregnancy showed that not only was there no benefit for the IL‐1R1^−/−^ mice, but their metabolic health worsened both during pregnancy and the postpartum period compared to their wild‐type counterparts (Plows et al., 2021). As nutrients can have relatively subtle effects on inflammation, they represent a less risky option. Indeed, studies presented in this review demonstrate the beneficial effects of anti‐inflammatory lipids and polyphenols.

These studies were not without limitations. Translatability is the biggest limitation, with many of these anti‐inflammatory compounds not comprehensively assessed in human RCT studies. Fish oil is the exception, with several RCT studies showing beneficial effects, albeit not necessary in relation to adiposity. Biochemical and anthropometric measures were extensively characterised; however, it is difficult to tease out any molecular insight. Indeed, the utility of animal studies in relation to molecular understanding is critical. These studies demonstrated some contradictory results, but it is possible that flaws in experimental design may be responsible for this with key factors such as sex‐specificity and treatment on control rather than HFD background presenting as problematic for appropriate translatability. Indeed, many of the studies examined utilised only male offspring, despite mounting evidence of differential effects between males and females in relation to metabolic health. Furthermore, some studies do not mention which sex was used and results may have been from pooled male and female samples, which may have masked key outcomes. Another key issue is lack of appropriate controls. There is evidence that intervention with anti‐inflammatory nutrients in a control setting may have detrimental effects, highlighting that a personalised approach should be adopted. It is therefore imperative that treatment with anti‐inflammatory nutrients is included in control diet settings. Therefore, it is imperative that the correct message on nutritional supplementation during pregnancy is formalised before it is delivered to the correct populations.

The early life period is a critical intervention window for the prevention of developmentally programmed adipose tissue dysfunction, and there is clear evidence that offspring from mothers living with overweight or obesity have higher risk of themselves developing cardiometabolic disease. Given the impact of obesity‐induced inflammation and disruption of key adipose tissue signalling pathways, anti‐inflammatory nutritional strategies represent a potential way to reduce inflammation and metabolic dysfunction in both the mother and her offspring. While many of these anti‐inflammatory nutraceuticals have yet to be comprehensively assessed in human studies, promising effects have been observed in animal studies. Deciphering the molecular mechanisms which help to shape healthy adipose tissue from early life will aid in uncovering nutritional strategies that can be targeted to the right individuals.

## AUTHOR CONTRIBUTIONS

Clare M. Reynolds and Michelle L. Kearns wrote and edited the manuscript. Both authors have read and approved the final version of this manuscript and agree to be accountable for all aspects of the work in ensuring that questions related to the accuracy or integrity of any part of the work are appropriately investigated and resolved. All persons designated as authors qualify for authorship, and all those who qualify for authorship are listed.

## CONFLICT OF INTEREST

There are no conflicts of interest.
